# Dietary Intake of Polish Female Soccer Players

**DOI:** 10.3390/ijerph16071134

**Published:** 2019-03-29

**Authors:** Hubert Dobrowolski, Dariusz Włodarek

**Affiliations:** Department of Dietetics, Faculty of Human Nutrition and Consumer Sciences, Warsaw University of Life Sciences (SGGW), 159c Nowoursynowska Str., 02-776 Warsaw, Poland; dariusz_wlodarek@sggw.pl

**Keywords:** diet, sport nutrition, women, needs fulfilling, energy expenditure

## Abstract

The aim of the study was to evaluate the energy expenditure and fulfillment of nutritional needs of female soccer players. Participants in this research were 41 professional soccer players from the three Polish female soccer league levels: Ekstraleague, I League and II League. The participants had their height and body mass measured. Total Energy Expenditure was measured by means of a SenseWear Pro3 Armband device. Data related to the food-intake energy values and the consumption of macro- and micronutrients were obtained through systematic recording of results, which was conducted over a three-day-long period at the start of the competitive season. The average age of the participants was 21 ± 5 years, the average height was 167.5 ± 5 cm, and the average body mass was 62.53 ± 9.8 kg. The average energy expenditure of the participants was 2811 ± 493 kcal/day, and their average energy intake was 1476 ± 434 kcal/day. The average consumption of carbohydrates, fats, and proteins was 199 ± 20.6, 47.3 ± 20.7, and 72.3 ± 24.2 g/day, respectively. There was a prevalence of inadequate intake of potassium, calcium, magnesium, iodine, vitamins D, E and B1, and folate in the diet of the examined group. The remaining micronutrients were consumed in the prescribed amounts by at least 50% of the examined group. The participants demonstrated low energy intakes, and consequently, low consumption of macronutrients and a large number of micronutrients.

## 1. Introduction

Soccer is one of the most popular sports in the world. As its popularity is still increasing, the number of both male and female players is on the rise, too. It is estimated that, in 2014, there were 30,145,700 women participating this sport, including 4,801,360 registered players [1]. Even though the number of female players is high, the International Federation of Association Football (Fédération Internationale de Football Association, FIFA) is committed to increasing the rates of female soccer players. 

One of the most important factors benefiting physical efforts is diet. A considerable body of research has demonstrated that an optimal dietary strategy influences the performance of, and recovery from, sporting activities [2]. A well-balanced diet, which is calculated to satisfy the increased energy demand of athletes, should provide the body with a sufficient amount of macro- and micronutrients, and guarantee maximal physical efficiency during training and competition [3]. Moreover, for people participating in competitive sports, proper nutrition constitutes one of main determinants of the achieved results during sports competitions [4], whereas the excess or deficit of some nutrients may negatively affect the results [5].

The same standards can be applied to soccer, since male and female soccer players also show special nutritional needs determined by the type of manual effort involved. Research shows that, on average, soccer players are capable of performing 726 different actions, moves, and turns during a match [6], whereas their capacity for fast high-intensity performance has a significant impact on the results of the games [7]. Research suggests that agility, strength, the ability to perform repetitive sprinting, and stamina are crucial success factors in soccer [8].

Considering the role of a well-balanced diet in athletes’ efforts, as well as the specificity of soccer, there was a need to conduct research in this field. So far, only a few studies have been carried out with the participation of professional female soccer players. There are also no studies comparing the diets of female soccer players at various league levels in different field positions in official matches. 

The aim of the study was the evaluation of the energy expenditure of female soccer players from the three soccer leagues, followed by an assessment of the fulfillment of energy and nutritional needs in the examined group.

## 2. Materials and Methods 

This research involved 41 professional female soccer players playing in different league-level clubs according to the Polish soccer league system: Ekstraliga (Ekstraleague, *n* = 14), I Liga (I League, *n* = 17) and II Liga (II League, *n* = 10), respectively, located near Warsaw. Details of the examined group are presented in Table 1. The research and all procedures were approved by the local Ethics and Scientific Research on Humans Commission (approval number: 24/2017, 19 June 2017). All participants or (if underage) their legal guardians consented to take part in the study, to have all measurements taken, and undergo various tests during the research.

Players were expected to meet the following requirements: be under 35 years old, be registered in the local Soccer Association, and take an active part in training sessions. Participants with long-term injuries (not permitted to train within the last 6 months), diagnosed with any long-term disease, and those who had failed to submit or submitted incomplete 3-day dietary food records were excluded from the study. 

Participants’ body mass and height were measured twice by means of a standard scale and stadiometer, accurate to within 0.1 kg and 0.1 cm, respectively. Measurements were taken in a fasting state before a training session. The final result was computed on the basis of the arithmetic average of the measurements. During body-mass measurements, participants were asked to only wear underwear; during height measurements, no footwear or socks were allowed. 

To assess the consumption of particular nutrients, the 3-day dietary food recording method was used. Since the weekly training schedule of the participants included most weekdays, they completed the recording on two training days and one day without training, during which there were no official league games. The players were asked to assess the volume of the food portions by means of an Album of Photographs of Food Products and Dishes (in Polish: Album fotografii produktów i potraw) [9] and the website ilewazy.pl [10]. The ilewazy.pl website is a free internet service that helps users assess the mass of a particular product. It includes a large picture database of products presented in various combinations (in cups, on plates, in the palm of a hand, etc.), which helps users assess the proper mass of consumed dishes and products. If the answers provided in the assigned charts of the 3-day dietary food records were unclear or illegible, the participants were asked further questions on the spot so as to ensure the maximum accuracy of responses relating to product consumption. The energetic value of food intake, as well as the ingredients of products and meals, were estimated by means of the Dieta 5.0 software (IŻŻ, Warsaw, Poland). Dieta 5.0 is a valuable application that calculates the intake of different nutrients, with bioavailability taken into account. 

To measure energy expenditure, the SenseWear Pro3 Armband device was used (Body Media Inc., Pittsburgh, PA, USA). The apparatus is equipped with five sensors: two skin galvanometers that measure the electrical conductance of skin, a skin temperature sensor, a heat-flux sensor that measures the rate in which the body dissipates heat relative to air temperature, and a 3-axial accelerometer to calculate the motion and intensity of physical activity, and the output of total energy expenditure resulting from the intensity of the physical activity. The structure of the device enables measurement reading, including total energy expenditure (TEE), activity energy expenditure (AEE), the average physical-activity level (PAL), the average METs (METs; one MET is the equivalent of aspirated oxygen in 3.5 mL O_2_/kg body mass/min), the time expenditure of different intensity activities (divided into five categories: <1.5, 1.5–3.0, 3.0–6.0, 6.0–9.0, and >9.0 METs), and relaxation-time expenditure, including resting and sleeping. Research conducted by means of this method is marked by high-accuracy measurements (TEE, AEE, NS, PAD, resting, sleeping) [11,12], repeatability (TEE, AEE) [11], and a low mean error rate (TEE, PAD) [13]. Participants wore armbands on the triceps of their right arm for at least 24 hours. The device was placed on participants’ arms for more than 30 minutes before the training session. Analysis of the records was completed by means of the SenseWear 8.1. software (Body Media Inc., Pittsburgh, PA, USA). 

The amount of energy per food intake was compared to the TEE results (obtained on a daily basis), and the energy-intake requirements described in the work of Brewer [14]. Fulfillment of the macronutrient norm was assessed on the basis the requirements listed by Burke et al. [15], Lemon [16], and Clark [17]. In compliance with the requirements of the Academy of Nutrition and Dietetics (AND), the Dietitians of Canada (DC), and the American College of Sports Medicine (ACSM) [2], the national recommendations were used [18] to assess the fulfillment of nutritional needs in terms of mineral components and vitamins. To assess an individual’s iron intake, the Qualitative Approach was used, and the z-score was calculated for a nutrient with an Estimated Average Requirement (EAR), according to the method described by the Institute of Medicine of the National Academies [19]. An approach was developed that statistically estimates the level of confidence that an individual’s usual intake is above an individual’s requirements [19].

Statistical analysis was conducted by means of the SPSS v. 20 software (IBM Corp., Armonk, NY, USA). To verify the normality of distribution, the Shapiro–Wilk test was used. The Student’s *t*-test (in the case of parametric values) and Wilcoxon’s test (in case of nonparametric values) were applied in order to compare the amount of nutrient consumption with the league level and the player’s position (goalkeeper, defender, midfielder, and striker). The correlation was based on Spearman’s test. The study’s defined significance level was set to α = 0.05. 

## 3. Results

The energy values of food intake, consumption of macronutrients, and fulfilment of nutritional needs concerning their consumption recommendations are presented in Table 2 and Table 3. The average energy value of the food intake was 1476 ± 434 kcal/day. A total of 95.1% of the examined female soccer players did not fulfil the daily energy demand assessed by means of armbands, and 97.6% of the players did not fulfil the energy demand assessed on the basis of the requirements provided in the work of Brewer [14]. The average consumption of carbohydrates, fats, and proteins was 199 ± 20.6, 47.3 ± 20.7, and 72.3 ± 24.2 g/day, respectively. There were no differences between the examined groups and the energy value of food intake (*p* > 0.05). There were also no differences between the groups and macronutrient intake (*p* > 0.05).

In each case, more than a half of the examined soccer players did not meet their macronutrient needs. No statistically significant differences in the macronutrient and energy demands between the groups (of different league levels) and the players’ positions were found (*p* > 0.05, Wilcoxon test). There were also no differences between the examined groups and the fulfilment of particular macronutrient and energy demands (*p* > 0.05, Wilcoxon test). 

Table 4 presents the consumption and fulfilment of nutritional demand for mineral components and vitamins.

The prevalence of inadequate intake in the examined group was high regarding the consumption of potassium, calcium, magnesium, iodine, vitamin B1, and folate in the dietary plan. The demand for sodium, phosphorus, and vitamins B2 and B6 was met by at least 80% of the examined group. Other micronutrients were consumed in the prescribed amounts by at least 50% of the examined female players. Research showed the statistical significance between consumption of iron (r = 0.393, *p* = 0.11, Spearman’s test) and copper (r = 0.322, *p* = 0.40, Spearman’s test) and the player’s position. The consumption rate of these components was higher for the center-forwards. Hence, the food intake of offensive players included more components than the food intake of the defensive players. No more statistically significant correlation between the consumption rate of mineral components/vitamins and the league level of the examined players, as well as the official position, was found. The usual intake of iron was adequate in 17 (41.5%) women, with a ≥70% probability of adequacy (z-score ≥ 0.5). A z-score of about 0.0 was observed in 14 (34.1%) women, which means that the probability of adequacy was about 50%. In addition, 24.4% of women had a high probability of inadequate iron intake (z-score ≤ −0.50, probability with an adequacy ≤30%). There was no statistically significant difference between leagues and the player’s positions (*p* > 0.05).

## 4. Discussion

To the best of our knowledge, ours is the first study of this kind conducted on Polish female soccer players. It is also the first study comparing the energy value of food intake of female soccer players with individual TEE assessed by means of armbands. Moreover, as far as we know, the present study is also the first describing and comparing the consumption of particular nutrients with the food intake between players of three league levels (Ekstraleague, I League, II League) and players of different positions (goalkeeper, defender, midfielder, striker). 

### 4.1. Energy

The average food-intake energy value of participants was 1476 ± 434 kcal/day, which amounted to 24.3 ± 8.9 kcal/kg body mass/day. Consumption was considerably lower than the results obtained by other authors. Martin et al. [20] in their research showed energy consumption of 1904 ± 366 kcal/day. In their work, Mullinix et al. [21] also showed higher energy values of food intake among female soccer players (2015 ± 19 kcal/day) than in the present research. In their research based on female soccer players, which was conducted before and after the season (including the method of three-day dietary food recording), Clark et al. [22] obtained results of 2290 ± 310 kcal/day (37 ± 5 kcal/kg body mass/day) and 1865 ± 530 kcal/day (30 ± 18 kcal/kg body mass/day), respectively. Both results are higher than those obtained in the present research. Abood et al. [23] obtained a food-intake energy value of 1969 ± 414 kcal/day, and Gravina et al. [24] of 2271 ± 571 kcal/day. Fogelholm et al. [25] assessed an energy intake of 8.97 ± 1.68 MJ/day (2142 ± 401 kcal/day). Moreover, Gibson et al. [26] obtained a higher energy value of food intake (2079 ± 460 kcal/day), even though their research was conducted on junior league players (aged 15.7 ± 7).

The energy-intake rate at this level was not only below the required values (24.3 ± 8.9 kcal/kg body mass/day vs. 47–60 kcal/kg body mass/day), but also below the obtained energy expenditure (2811 ± 493 kcal/day; 45.7 ± 9 kcal/kg body mass/day). An energy shortage of 1335 ± 708 kcal on average in the case of applying an Armband device, and 1462 ± 703 kcal in the case of applying the standards described in the research of Brewer [14] fell considerably below the expected average, which cannot be ignored. Only one person met the demand of 47 kcal/kg body mass/day, which is the marginal norm of 47–60 kcal/kg body mass/day specified in the requirements. Only the energy value of the food intake of two players exceeded the energy expenditure assessed by means of the armbands. Inadequate energy intake may lead to negative consequences, such as chronic tiredness or a higher risk of infections and diseases. Some complications may also be found in the cardiovascular, digestive, endocrine, reproductive, skeletal, renal, or central nervous systems [27], as well as many other conditions resulting in a decrease of exercise capacity, such as body-mass decrease, including potential muscle-mass decrease [20]. Deficient energy intake may lead to the development of the Relative Energy Deficiency in Sport syndrome (RED-S), which may produce serious health problems (impairment of immunological functions, menstrual-function disorder, osteoporosis, disorders of the endocrine, metabolic, hematological, psychological, gastrointestinal, and cardiovascular systems, and growth disorders) and affect exercise capacities (increased injury risk, decreased training response, impaired judgment, decreased motor coordination and concentration, irritability, depression, decreased glycogen supplies, and decreased muscle strength and stamina) [28]. It is also worth noting the risk of incidence of the so-called Female Athlete Triad syndrome, in which such disorders as amenorrhoea and osteoporosis, caused by low energy availability, is present [27,28]. Many sportswomen intaking less energy than required complained of tiredness, higher incidence of injuries, irritability, and reduced performance [29]. 

It is difficult to account for such low results concerning food consumption in our research. Some authors suggest that regular food-intake rates among sportswomen might have been considerably underestimated during the note-taking process. However, other research shows that this underestimation is not higher than in the case of men [30]. The lack of differences in terms of underestimation levels between men and women in completed records does not exclude the possibility of underestimation in the case of the examined group. Another reason for obtaining such low food-intake energy values was the commencement of the soccer season, which resulted in daily-schedule alterations as well as increased stress levels caused by the pressure to attend official events. However, considering the normal BMI value of the participants, it is unlikely that the food-intake energy value of the examined players is typical for them, and that most of their diet is characterized by such low energy intake. Only three participants had a BMI of <18.5 kg/m^2^, while the remaining ones had healthy body-mass values. One should remember that the most important determinant of energy intake in a diet that is adequate to needs is body mass. Hence, one may claim that the obtained inadequate energy value of the diet was probably the result of underestimation of the food-intake amount, or a temporary decrease in food consumption. 

### 4.2. Macronutrients

In the present study, the number of macronutrients in players’ diets was low, which resulted from low food-intake energy values. Fat intake levels were by far the closest to the expected standards. The fat intake of 16 study participants exceeded 30% of diet energy. Nevertheless, it is worth noting that fat intake in the examined group was 47.3 ± 20.7 g/day, whereas on average the norm (<30% of diet energy) was <49.2 ± 14.5 g/day. The fact that the group’s average consumption was close to marginal values suggests that the vast majority of research participants were at least close to 30% of diet energy. It needs to be pointed out that this is the upper limit of fat levels in a diet (30% of diet energy), which the players should not exceed because it may result in insufficient intake of proteins and carbohydrates, which play a main role in players’ dietary plan. A high fat intake almost certainly eliminates the possibility of proper protein and carbohydrate intake when the energy value of the participants’ dietary plan is too low. 

The lower limit of the protein-intake norm (1.4 g/kg body mass/day) was achieved by 11 participants, six of which exceeded the upper limit of the recommended values (1.7 g/kg body mass/day). The average intake dose for the examined group, 1.18 ± 0.45 g/kg body mass/day, was above the required 0.8 g/kg body mass/day, which allows one to maintain proper bodily functions, but may negatively affect performance [31]. Despite these facts, it should be noted that 19.5% of female players did not exceed the 0.8 g/kg body mass/day dose, which may not only lead to a decline in results, but also pose a danger to health. Moreover, Tipton and Wolfe [32] concluded that the recommended protein-consumption level for athletes is 1.2–1.7 g/kg body mass/day. Dolins [33] also showed that, for sportswomen, a 1.2 g/kg body mass/day dose is sufficient to maximize performance potential. Boisseau et al. [34] claim that, for male soccer players, the protein EAR is 1.2 g/kg body mass/day. Even though this research was conducted on men, it is legitimate to assume that the norms of proteins intake for both genders are the same [35]. With regard to protein intake in the examined group in terms of this level of demand, it can be concluded that the number of proteins in participants’ food intake (1.18 ± 0.45 g/kg body mass/day) was only slightly below the recommended values. Hence, without examination of nitrogen balance, it is difficult to establish whether the protein-intake level was below the recommended values due to the absence of the recommended average protein-intake levels. Nonetheless, it may be assumed that 43.9% of the examined group consumed the prescribed protein amount. The balanced consumption of protein is very important for muscle protein synthesis, exertional regeneration, growth stimulation, or body composition. 

The amount of carbohydrates in athletes’ food intake is crucial. Carbohydrates are a source of energy during physical efforts, and they influence their effectiveness. A carbohydrate intake of 199 ± 20.6 g/day is a critically low level. Only three participants fulfilled the demand for the 5 g/kg body mass/day norm of carbohydrate intake, and only one participant exceeded 7 g/kg body mass/day. Economos et al. [36] recommended consuming at least 6 g/kg body mass/day of carbohydrates when the energy intake is <45 kcal/kg body mass/day. This criterion was not met, since carbohydrate intake in the examined group was 3.3 ± 1.2 g/kg body mass/day on average. Carbohydrate intake at such a low level may lead to a deterioration of post-exercise muscle glycogen resynthesis and, as a result, a deterioration of participants’ physical efficiency. Moreover, low carbohydrate intake increases injury risk [37]. A fiber intake of 14.4 g/day (median) is below the average dose specified in Polish norms (78% of the examined female soccer players consumed less than 20 g/day of fiber in their diet). 

Similar results (fat intake of about 30% diet energy, protein intake of about 1.2 g/kg body mass/day and a carbohydrate intake considerably below the recommended intake values) were obtained by Martin et al. However, even in their research, carbohydrate intake was not as low as the present study indicates (3.3 ± 1.2 g/kg body mass/day vs. 4.1 ± 1 g/kg body mass/day) [20]. Fat levels of 30% of diet energy are often indicated by other researchers [20,21,22,26]. Nevertheless, there is research conducted on female soccer player in which fat intake is both lower [22] and higher [24] than recommended. Research by Gibson et al. [26] showed that protein-intake rates met the recommended standards, as did research by Clark et al. [22], but only before the beginning of the season. After the season, intake was lower than the recommended values. Martin et al. [20] demonstrated that intake-level results are compatible with the results of this study. Mullinix et al. [21] showed intake equal to 1.3 g/kg body mass/day, which is lower than recommended but higher than presented in our research. Carbohydrate intake was lower than recommended [20,21,22] but, in research by Clark et al., carbohydrate intake was too low after the soccer season and, within normal specifications, before the season, as was in the case of protein intake. Gibson et al. [26] also showed that intake levels were within the normal limits. Fiber intake in the present study was lower than recommended and lower than in other studies [24,26]. 

### 4.3. Micronutrients

Insufficient consumption of mineral components and vitamins was the result of the low energy value of food intake. Only sodium, phosphorus, iron, zinc, copper, and vitamins A, C, B2, PP, B6, and B12 were consumed in amounts higher than the EAR norm by more than a half of the examined group. Other mineral-component content (potassium, calcium, magnesium, iodine) and vitamin content (B1, folate) in food intake were too low for all examined women. 

Consumption of vitamin D was at a critically low level. Even though supplementation with this vitamin increased the muscle strength and balance of older-people populations [38], conducted research on athletes has not proven a positive influence on physical efficiency so far [39,40]. However, there are studies showing that vitamin D may have a positive influence on physical efficiency for people who have a deficiency in these vitamins [2,41]. Vitamin D is also endogenously produced as a consequence of UVB radiation. Considering that the examined group consisted of female soccer players exposed to this radiation, as well as the examination period (the end of the summer season), one may assume that vitamin D concentration in the blood might be at proper levels. As research suggests, during this period, concentration of 25-hydroxyvitamin D in serum is highest at the end of the summer season and at the beginning of the autumn season [41]. However, this remains unconfirmed without proper research and defining the concentration of vitamin D in serum. This confirmation is important, especially because evaluation was made with an adequate-intake (AI) recommendation. Failure to meet the consumption threshold at this level does not necessarily mean that intake is insufficient. Nevertheless, while calling attention to the low calcium amount in the food intake of the female soccer players, and the increased risk of skeletal-system problems caused by low food-intake energy values, it is necessary to emphasize the proper intake of this vitamin, for example, via supplementation, which is vital for maintaining healthy bones [42].

Even though thiamin intake in the examined group was low, its short-term deficiency does not affect physical efficiency [41]. However, inadequate folate intake does influence physical efficiency. As was demonstrated, folate and vitamin B12 deficit impaired circuit training [43]. Moreover, it has to be highlighted that all participants were of childbearing age. Although in terms of manual-effort effectiveness it is not significant, insufficient folic acid intake impairs proper fetus growth and leads to neural-tube defects. The increase of folate consumption is advisable not only with regard to physical efficiency, but also in terms of health and procreational aspects. 

A total of 80.5% of the examined group consumed potassium in amounts lower than the EAR. Potassium is a very important diet component since it regulates intra- and extracellular fluid volume, and it is also involved in nerve impulses and skeletal muscle-cramp conduction [44]. As research shows, a 3000 mg potassium daily intake allows the body to maintain a positive balance in the case of daily sweat loss of 3 liters [45]. The occurrence of potassium deficit in the body is a very rare phenomenon and it is more often caused by vomiting, diarrhea, or diuretics [44].

Calcium is important in a sportsperson’s diet not only for maintaining healthy bones but also because it affects muscle contraction, blood coagulation, neural impulses, protein usage, and cell signaling [46]. Nonetheless, the main negative consequence of low calcium intake, especially connected with low vitamin D intake, is a decrease of bone-mineral density and, in turn, an increased risk of bone fractures [42]. Calcium intake among participants was very low, which, combined with low vitamin D intake, may result in an increased risk of injuries during training sessions and games. A total of 82.9% of players had inadequate calcium intake. 

A large proportion of female soccer players did not meet the required levels of magnesium intake. However, it is also worth noting that magnesium deficit is rarely caused by insufficient consumption of this component in a diet, but rather it is a consequence of some diseases [46]. Magnesium is a significant component of every athlete’s diet. Its deficit may lead to myasthenia, cramps, neuromuscular dysfunctions, immunosuppression [46], and it may also have an impact on physical-efficiency decrease [47]. Hence, balancing magnesium intake in keeping with the prescribed values is highly recommended for the examined group. 

Undoubtedly, iron deficit affects the physical efficiency of athletes [48]. However, it affects women before menopause more than men, which is due to iron loss during the menstrual cycle [49]. The main functions of iron are as a hemoglobin component, in oxygen transport from the lungs to tissue, and its deficit leads to a decrease in physical efficiency [50]. Research has shown that the deficit is a common problem among female soccer teams [51]. DellaValle in her study highlighted the need for constant control of the hemoglobin and ferritin levels of female athletes who are at risk of iron deficit at the beginning and during the training period, as well as the necessity of proper dietary recommendations and iron supplementation of athletes who showed a deficit [52]. Even though the vast majority of participants met iron-intake demand, there is still a great part of the examined players whose iron intake was very low. Ten study participants had a high probability of inadequate intake, and fourteen only had a 50% probability of adequate intake. Unfortunately, since no morphology and ferritin tests were done, we are not able to confirm the levels of iron deficit in the examined group. Nevertheless, given that iron deficiency is a common phenomenon, this appears to be highly possible. 

Research based on female soccer players conducted by other scholars has also indicated a deficit of microcomponents in food intake. Mullinix et al. using similar methods showed insufficiency of vitamins D, E, and folate (to a lesser degree), magnesium, zinc, and, to a lesser degree. calcium and phosphorus [21]. Authors did, however, compare their results with the Dietary Reference Intake (DRI) provided by the U.S. Institute of Medicine, which is based on RDA and AI norms, while we used the national EAR and AI. Gibson et al., in turn, pointed to the insufficiency of pantothenic acid, folate, calcium, and magnesium in the diet of the vast majority of the participants, and insufficiency of vitamins D and E in the diet of all the participants [26]. These authors, like us, used EAR and AI norms, which, in our opinion, appears to be best method. Research by Clark et al. has demonstrated inadequate levels of chromium, copper, magnesium, zinc, folic acid, biotin, pantothenic acid, and vitamin D intake, both before and after the season, as well as a low intake of iron, selenium, and vitamins E, C, and B6 after the season [22]. The results of these studies were compared to the 1998 RDA norms and 2001 DRIs as the RDA and AI norms when calculating the prevalence of inadequate intakes, while we used EAR and AI norms. We need to point out that the RDA norms used in other studies are less correct than EAR norms to assess the fulfillment of needs in groups like soccer teams. Thus, it is also hard to compare our results with results presented by other authors who used different norms. While comparing the obtained results to the results of other scholars, one may claim that vitamin E intake in the examined group was acceptable. Analysis of our research against the background of other studies also shows a relatively common occurrence of calcium, magnesium, vitamin D, and folic acid insufficiency among female soccer players. Hence, it is important to make female players and coaches aware of the consumption of food groups that include these components. 

Despite using properly selected methods, some limitations of this study must be indicated. First of all, energy-expenditure measurement was made over a 24-hour period. Although we were assured by coaches that, during the entire week when measurements were taken, training programs were extremely similar, which indicates that energy expenditure was also similar, our results cannot be taken as the normal energy expenditure of elite female soccer players. It is important to note that energy expenditure can be different in training microcycles and strongly depends on the training program in that week and competitive season. Our studies were also carried during the beginning of the competitive season. Changes in lifestyle and transitions between training macrocycle phases could impact athletes’ energy intakes. This could explain the low energy intake compared to energy expenditure. This is, however, an important element of our study. This indicates to trainers and sport nutritionists the risk of such changes, which may affect physical performance during the first matches in a competitive season. Finally, our sample size could be too small for calculating inadequate intake. Studies with more participants are recommended.

## 5. Conclusions

In conclusion, our research showed low energy intake and, consequently, of macrocomponents, by female soccer players. In most cases, they consumed an insufficient amount of carbohydrates. Protein intake was also inadequate. Vitamin B1, folate, and mineral components such as potassium, calcium, magnesium, and iodine were consumed by most of the participants in smaller amounts than recommended in the national recommendations. The prevalence of these nutrient inadequacies is likely to be high in examined group. Vitamins E and D were consumed below the adequate intake levels. Low intake can, however, be the result of lifestyle changes. Future research should concentrate on the intake of these components by other sportswomen and the consequences of deficits in the diet of female soccer players. The present study, as well as other related studies, show that determining the amounts of particular vitamins and mineral components required for professional female soccer players to prevent deficits appears to be well-founded.

## Figures and Tables

**Table 1:** Characteristics of the research group.

Characteristic	Ekstraleague (mean ± SD min–max median)	I League (mean ± SD min–max median)	II League (mean ± SD min–max median)	Overall (mean ± SD min–max median)
**Age (years)**	24 ± 5 15–31 25	19 ± 4 13–27 19	20 ± 3 17–25 19	21 ± 5 13–31 20
**Height (cm)**	168.1 ± 6.2 156–180 167.5	167.1 ± 4.3 160–176 167	167.5 ± 4.6 162–175 167	167.5 ± 5 156–180 167
Body mass * (kg)	62.19 ± 6.62 54.1–77.2 59.3	64 ± 12.67 49.1–94 59.2	60.47 ± 8.45 49.3–77.2 58.5	62.53 ± 9.8 49.1–94 59.2
BMI * (kg/m^2^)	21.99 ± 1.83 19.62–25.29 21.86	22.81 ± 3.66 19.62–25.29 21.8	21.52 ± 2.56 18.03–25.21 21.34	22.22 ± 2.86 17.61–31.16 21.53

* Body mass and BMI do not have a normal distribution (*p* < 0.05, Shapiro–Wilk test).

**Table 2:** Food intake, energy fulfillment, and macronutrient demand.

Component	Intake (min–max)	Intake (median/mean ± SD)	Demand (median/mean ± SD)	Percentage of People Scoring Below Required Norms
**Energy (kcal)**	822–2606	1476 ± 434	2811 ± 493 *	95.1%
**Carbohydrates (g)**	79.2–359	199 ± 20.6	296–414 ^a^	92.7%
**Fats (g)**	9.3–95.6	47.3 ± 20.7	49.2 ± 14.5 ^b^	61%
**Proteins (g)**	10.6–135	72.3 ± 24.2	82.9–100.6 ^c^	73.2%
**Fiber (g)**	5.1–52.9	14.4	20–40 ^d^	78%

* Data obtained by means of the ArmBands. a—carbohydrates demand according to Burke et al. [15] (5–7 g/kg body mass/day). b—fats demand according to Clark [17] (<30% of diet energy). c—proteins demand according to Lemon [16] (1.4–1.7 g/kg body mass/day). d—fiber demand according to Polish norms [18] (20–40 g/day).

**Table 3:** Food intake, energy fulfillment, and macronutrient nutritional needs in terms of body-mass kilograms.

Component	Intake (mean ± SD)	Intake (min–max)	Demand	Percentage of People Scoring Below the Required Norms
**Energy** **(kcal/kg body mass/day)**	24.34 ± 8.9	10.65–53.07	47–60 ^a^	97.6%
**Carbohydrates** **(g/kg body mass/day)**	3.28 ± 1.2	1.39–7.09	5–7 ^b^	92.7%
**Proteins** **(g/kg body mass/day)**	1.18 ± 0.44	0.21–2.16	1.4–1.7 ^c^	73.2%
**Fats** **(g/kg body mass/day)**	0.78 ± 0.39	0.19–1.95	- ^d^	- ^d^

a—energy demand according to Brewer [14]; b—carbohydrates demand according to Burke et al. [15]; c—proteins demand according to Lemon [16]; d—lack of requirements as to fats intake in terms of body-mass kilograms.

**Table 4:** Mineral-component and vitamin intake, and demand fulfillment.

Component	Intake (min–max)	Intake (median/mean ± SD)	Demand (EAR or AI)	Prevalence of Inadequate Intake
Sodium (mg)	696–6246	2583 ± 1077	1500	12.2%
Potassium (mg)	960–4497	2584 ± 878	3500	80.5%
Calcium (mg)	146–1440	646 ± 290	800/1100 *	82.9%
Phosphorus (mg)	255–1964	1165 ± 357	580/1050 *	17.1%
Magnesium (mg)	66.7–616	243.9	255/300 *	58.5%
Iron (mg)	2.18–23.7	8.8	8	-
Zinc (mg)	1.33–14.8	8.44 ± 2.77	6.8/7.3 *	26.9%
Copper (mg)	0.28–2.14	0.96 ± 0.39	0.7	24.4%
Iodine (μg)	6.53–236	86.37	95	53.6%
Vit. A (μg)	58.4–3239	651.8	490/500 *	34.1%
Vit. E (mg)	2.77–16.5	6.4	8 **	- **
Vit. D (μg)	0.18–11.5	1.69	15 **	- **
Vit. C (mg)	14.4–371	73.56	55/60 *	41.5%
Vit. B1 (mg)	0.33–3	0.86	0.9	56.1%
Vit. B2 (mg)	0.3–2.86	1.49 ± 0.55	0.9	12.2%
Vit. PP (mg)	2.3–39.9	17.2 ± 8.14	11	24.4%
Vit. B6 (mg)	0.6–3.25	1.7 ± 0.65	1/1.1 *	19.5%
Vit. B12 (μg)	0.33–6.13	2.9 ± 1.38	2	29.3%
Folate (μg)	58.9–462	224 ± 95.8	320/330 *	85.4%

Vitamin and mineral-component demand according to national recommendations [18]. EAR—Estimated Average Requirement. * The norm relative to the participant’s age. ** Adequate Intake (AI).
